# Prevalence, predictors and perinatal outcomes of peri-conceptional alcohol exposure - retrospective cohort study in an urban obstetric population in Ireland

**DOI:** 10.1186/1471-2393-11-27

**Published:** 2011-04-11

**Authors:** Aoife Mullally, Brian J Cleary, Joe Barry, Tom P Fahey, Deirdre J Murphy

**Affiliations:** 1 Academic Department of Obstetrics & Gynaecology, Coombe Women and Infants University Hospital & Trinity College Dublin, Dublin 8, Republic of Ireland; 2 School of Pharmacy, Royal College of Surgeons in Ireland, Dublin, Republic of Ireland; 3 Department of Public Health and Primary Care, Trinity College Dublin, Republic of Ireland; 4 HRB Centre for Primary Care Research, Department of Family Medicine and General Practice, Royal College of Surgeons in Ireland, Dublin, Republic of Ireland

## Abstract

**Background:**

Evidence-based advice on alcohol consumption is required for pregnant women and women planning a pregnancy. Our aim was to investigate the prevalence, predictors and perinatal outcomes associated with peri-conceptional alcohol consumption.

**Methods:**

A cohort study of 61,241 women who booked for antenatal care and delivered in a large urban maternity hospital between 2000 and 2007. Self-reported alcohol consumption at the booking visit was categorised as low (0-5 units per week), moderate (6-20 units per week) and high (>20 units per week).

**Results:**

Of the 81% of women who reported alcohol consumption during the peri-conceptional period, 71% reported low intake, 9.9% moderate intake and 0.2% high intake. Factors associated with moderate alcohol consumption included being in employment OR 4.47 (95% CI 4.17 to 4.80), Irish nationality OR 16.5 (95% CI 14.9 to 18.3), private health care OR 5.83 (95% CI 5.38 to 6.31) and smoking OR 1.86 (95% CI 1.73 to 2.01). Factors associated with high consumption included maternal age less than 25 years OR 2.70 (95% CI 1.86 to 3.91) and illicit drug use OR 6.46 (95% CI 3.32 to 12.60). High consumption was associated with very preterm birth (<32 weeks gestation) even after controlling for socio-demographic factors, adjusted OR 3.15 (95% CI 1.26-7.88). Only three cases of Fetal Alcohol Syndrome were recorded (0.05 per 1000 total births), one each in the low, moderate and high consumption groups.

**Conclusions:**

Public Health campaigns need to emphasise the importance of peri-conceptional health and pre-pregnancy planning. Fetal Alcohol Syndrome is likely to be under-reported despite the high prevalence of alcohol consumption in this population.

## Background

Many women continue to consume alcohol in pregnancy despite an increasing body of evidence suggesting harm to the fetus. A recent Australian study reported that only 41% of pregnant women abstained from alcohol throughout each trimester [1]. The number of women who drink alcohol early in the first trimester before recognition of pregnancy is likely to be even higher. Ascertaining the true prevalence and extent of alcohol consumption during pregnancy is difficult as under-reporting is common and women's understanding of what constitutes a standard "drink" or "unit" may differ from person to person. Making comparisons between studies in different countries is also difficult due to differences in drinking patterns and different alcohol content of drinks and units [2].

Studies linking alcohol consumption with first trimester miscarriage have been inconclusive [2-4] but a dose-related increase in second-trimester miscarriage has been reported [5]. Various studies have associated moderate to heavy alcohol consumption with both preterm birth and low birth weight [6,7]. However, the results are often inconsistent and the majority of studies have shown no significant associations when socio-demographic confounders are taken into consideration. The estimated incidence of Fetal Alcohol Syndrome (FAS) in Europe is 0.4 per 1000 live births [8]. Children with FAS may be only a small proportion of the total number of children affected by alcohol-induced effects including neuro-behavioural deficits, fetal alcohol spectrum disorder and alcohol related birth defects. A number of studies have reported a relationship between alcohol intake in pregnancy and behavioural and cognitive deficits in childhood and adolescence [9-11]. Binge-drinking in pregnancy is also of concern, especially as this pattern of drinking has increased in the female population and is associated with unplanned pregnancy [12].

The advice from the Department of Health and Children in Ireland is that alcohol should be avoided during pregnancy. This is similar to the advice by the Surgeon General in the United States - that pregnant women or women who may become pregnant should abstain from alcohol. Since 2007, the Chief Medical Officer of the United Kingdom has also advised that women who are pregnant or are trying to conceive should avoid alcohol. However, in 2008, the National Institute for Health and Clinical Excellence (NICE) in the United Kingdom issued the following guidance: "If women choose to drink alcohol during pregnancy they should be advised to drink no more than one to two units once or twice a week. Although there is uncertainty regarding a safe level of alcohol consumption in pregnancy, at this low level there is no evidence of harm to the unborn baby" [13]. This conflicting information is likely to be confusing for women who may wish to continue "social" drinking in pregnancy.

The aim of this study is to investigate the prevalence, socio-demographic predictors and perinatal outcomes associated with alcohol consumption in the peri-conceptional period using a large cohort of women booking for antenatal care and delivering in a Dublin maternity hospital.

## Methods

A retrospective cohort study was carried out using the electronic booking records of women with singleton pregnancies who delivered in a large Dublin maternity hospital between 2000 and 2007. The data from the booking interview was linked to the electronic delivery record and neonatal records with information on the infant up until first hospital discharge. Information on peri-conceptional alcohol consumption (prior to pregnancy and up until the pregnancy was confirmed) was recorded electronically using a computerised antenatal questionnaire administered by trained midwives at the patient's first antenatal visit, which usually occurred at about 12 weeks' gestation. Women were asked two questions at the time of booking; 1) whether they drink alcohol and 2) how many units of alcohol they drink per week. Women who described themselves as complete abstainers from alcohol were described as "never" drinkers. For women who reported drinking alcohol, levels of consumption were recorded in the following bands: 0-5 units per week (this group included very occasional drinkers who may have had no alcohol intake some weeks but did drink on occasions), 6-11 units per week, 11-20 units per week and >20 units per week. For the analyses subjects were divided into four groups: never drinkers, drinkers consuming 0-5 units per week (similar to the NICE recommendation for occasional alcohol intake and termed low alcohol intake), drinkers consuming 6-20 units per week (in excess of the NICE recommendation and termed moderate alcohol intake) and drinkers consuming greater than 20 units per week (excessive alcohol intake for women and termed high alcohol intake).

In addition to alcohol consumption, information on the following maternal characteristics was extracted from the electronic records: maternal age, marital status, socioeconomic group, nationality, public or privately funded antenatal care, parity, planned pregnancy, gestation at booking, smoking and illicit drug use and referral to a social worker. Maternal age was divided into the following bands: <20 years, 20-24 years, 25-29 years, 30-34 years, 35-39 years and >40 years. Socioeconomic groups were classified as professional/manager/employer, home duties, non-manual, manual, unemployed and non-classifiable. Nationality was recorded as either Irish or non-Irish and further sub-divided by region into Western Europe, Asia/Middle East, Eastern Europe, Africa, South America, North America, Australia & New Zealand. Gestational age at booking was divided into <12 weeks, 12-20 weeks and >20 weeks. Smokers were defined as women who were current smokers at the time of attendance at their first antenatal visit. Illicit drug users were defined as women who had ever used illicit drugs.

Perinatal outcome measures included gestational age at delivery, live birth or stillbirth, birth weight, infant gender, infant's condition at birth including Apgar scores at 1 and 5 minutes, admission to the special care baby unit, any suspected congenital abnormalities and whether resuscitation was required. Detailed data on the neonate were extracted on infants admitted to the neonatal unit including details on ventilation, suspected neonatal abnormalities including Fetal Alcohol Syndrome (FAS), genetic testing and neonatal death. Every woman had an ultrasound scan at the booking antenatal visit. Gestational age was estimated from the calculation based on first day of the last menstrual period but the booking ultrasound scan estimate was preferred if the dates were uncertain or there was a discrepancy of more than seven days. Preterm birth was defined as the birth of a live baby at less than 37 weeks gestation. Very preterm birth was defined as the birth of a live baby at less than 32 weeks gestation. Low birth weight was defined as weighing less than 2500 g and very low birth weight as less than 1000 g. Small for gestational age was defined as a birth weight less than the 10^th ^percentile using individualised birth rate ratios (corrected for maternal height and weight, parity, infant sex, ethnicity and gestation) http://www.gestation.net. Stillbirth was defined as delivery of a baby showing no signs of life at or after 24 weeks gestation.

The analyses were performed using the Statistical Package for Social Sciences (SPSS version 15). Descriptive statistics were used to characterise the study subjects by category of alcohol intake. Comparisons were made between the different alcohol intake groups to identify socio-demographic predictors of peri-conceptional alcohol intake. Logistic regression analyses were performed to measure the association between alcohol exposure and adverse perinatal outcomes. The "never" category was chosen as the comparator for each of the analyses as this was unlikely to be biased by under-reporting and represented a group where we could be certain that there was no alcohol exposure in the peri-conceptional period even for women with uncertain dates. Further comparisons were made adjusting for potential confounding factors including maternal age, single marital status, socio-economic status, nationality, private health insurance, nulliparity, unplanned pregnancy, late booking, smoking, and illicit drug use. These factors were chosen because of their known or possible association with perinatal outcome. Results are reported as proportions, odds ratios (OR) and 95% confidence intervals (CI).

The study received the approval of the Coombe Women and Infants University Hospital's research ethics committee: Study No. 22-2009.

## Results

A total of 61,241 antenatal booking and delivery records were available for analysis with very few missing responses for individual data items. The characteristics of the women in the cohort in relation to alcohol intake are presented in Table 1. The demographic characteristics of the women were similar to other urban populations in Ireland based on annual maternity statistics. Of the 61,241 women in the cohort, 49,628 (81%) reported alcohol consumption during the peri-conceptional period; 43,455 (71%) with low intake (an average of 0-5 units per week), 6,059 (9.9%) with moderate intake (6-20 units per week) and 114 (0.2%) with high intake (>20 units per week). A total of 11613 women (19%) reported that they never drank alcohol. Factors associated with moderate alcohol intake compared to no alcohol consumption included single marital status OR 1.98 (95% CI 1.85 to 2.11), being in employment OR 4.47 (95% CI 4.17 to 4.80), Irish nationality OR 16.5 (95% CI 14.9 to 18.3), private health care OR 5.83 (95% CI 5.38 to 6.31), nulliparity OR 3.30 (95% CI 3.10 to 3.52) and smoking OR 1.86 (95% CI 1.73 to 2.01). Factors associated with high intake compared to no alcohol consumption included maternal age less than 25 years OR 2.70 (95% CI 1.86 to 3.91), single marital status OR 11.80 (95% CI 7.28 to 19.2), Irish nationality OR 13.40 (95% CI 7.01 to 25.8), nulliparity OR 4.59 (95% CI 3.09 to 6.82), unplanned pregnancy OR 5.51 (95% CI 3.57 to 8.51), smoking OR 10.90 (95% CI 7.25 to 16.40) and illicit drug use OR 6.46 (95% CI 3.32 to 12.60). The risk factors for alcohol intake are summarised in Table 2.

**Table 1:** Characteristics of women according to alcohol intake peri-conceptionally

Alcohol intake	Never n = 11613 (%)	0 - 5 units n = 43455 (%)	6 - 20 units n = 6059 (%)	>20 units n = 114 (%)
Maternal age				
<20 years	649 (5.6)	2082 (4.8)	290 (4.8)	18 (15.8)
20-24 years	2031 (17.5)	5745 (13.2)	962 (15.9)	33 (28.9)
25-29 years	3463 (29.8)	9924 (22.8)	1275 (21.0)	28 (24.6)
30-34 years	3374 (29.1)	15059 (34.7)	2077 (34.3)	20 (17.5)
35-39 years	1746 (15.0)	9015 (20.7)	1224 (20.2)	11 (9.6)
>40 years	350 (3.0)	1630 (3.8)	231 (3.8)	4 (3.5)

Single Marital status	3151 (27.1)	14615 (33.6)	2567 (42.4)	91 (79.8)

Socioeconomic group				
Professional/Manager/Employer	1596 (13.7)	10893 (25.1)	1838 (30.3)	9 (7.9)
Home duties	3763 (32.4)	8193 (18.9)	763 (12.6)	9 (7.9)
Non-manual	2207 (19.0)	14935 (34.4)	2194 (36.2)	36 (31.6)
Manual	414 (3.6)	1948 (4.5)	248 (4.1)	11 (9.6)
Unemployed	2856 (24.6)	5403 (12.4)	695 (11.5)	37 (32.4)
Non-classifiable	770 (6.6)	2069 (4.8)	321 (5.3)	12 (10.5)

Irish Nationality	4953 (42.7)	37326 (85.9)	5519 (91.1)	103 (90.4)

Nationality - Region				
Western Europe	5411 (46.6)	39932 (91.9)	5862 (96.7)	109 (95.6)
Asia/Middle East	1852 (15.9)	377 (0.9)	10 (0.2)	0 (0.0)
Eastern Europe	1250 (10.8)	1151 (2.6)	13 (0.2)	0 (0.0)
Africa	2795 (24.1)	844 (1.9)	12 (0.2)	2 (1.8)
South America	58 (0.5)	77 (0.2)	2 (0.03)	0 (0.0)
North America	42 (0.4)	279 (0.6)	41 (0.7)	1 (0.9)
Australia & New Zealand	8 (0.1)	132 (0.3)	15 (0.2)	1 (0.9)

Private Health Care	1154 (9.9)	12087 (27.8)	2382 (39.3)	4 (3.5)

Nulliparous	3725 (32.0)	17719 (40.8)	3692 (60.9)	78 (68.4)

Unplanned pregnancy	4152 (35.8)	14750 (33.9)	2368 (39.1)	85 (74.6)

Gestation at booking				
<12 weeks	1455 (12.5)	8002 (18.4)	1250 (20.6)	15 (13.2)
12-20 weeks	5912 (50.9)	26260 (60.4)	3588 (59.2)	62 (54.4)
>20 weeks	2651 (22.8)	4349 (10.8)	460 (7.6)	12 (10.5)

Smoked during pregnancy	2102 (18.1)	9917 (22.8)	1776 (29.3)	80 (70.2)

Illicit drug use (ever)	171 (1.5)	311 (0.7)	53 (0.9)	10 (8.8)

Social worker referral	2471 (21.3)	5025 (11.6)	753 (12.4)	45 (39.5)

**Table 2:** Risk factors for alcohol intake peri-conceptionally

Alcohol intake^i^	Low (0 - 5 units) Odds Ratio (95% CI)	Moderate (6 - 20 units) Odds Ratio (95% CI)	High (>20 units) Odds Ratio (95% CI)
Maternal age <25 years	0.73 (0.70-0.77)	0.88 (0.81-0.94)	2.70 (1.86-3.91)
Single marital status	1.35 (1.29-1.42)	1.98 (1.85-2.11)	11.80 (7.28-19.2)
In employment	3.21 (3.07-3.35)	4.47 (4.17-4.80)	1.69 (1.16-2.47)
Irish Nationality	8.91 (8.50-9.34)	16.5 (14.9-18.3)	13.40 (7.01-25.8)
Private Health Insurance	3.47 (3.26-3.70)	5.83 (5.38-6.31)	0.33 (0.12-0.90)
Nulliparous	1.46 (1.40-1.52)	3.30 (3.10-3.52)	4.59 (3.09-6.82)
Unplanned pregnancy	0.91 (0.87-0.95)	1.14 (1.07-1.22)	5.51 (3.57-8.51)
Late booking (>20 weeks)	0.38 (0.36-0.40)	0.28 (0.25-0.31)	0.40 (0.21-0.72)
Smoked during pregnancy	1.33 (1.26-1.40)	1.86 (1.73-2.01)	10.9 (7.25-16.4)
Illicit drug use (ever)	0.48 (0.40-0.58)	0.59 (0.43-0.80)	6.46 (3.32-12.6)

^i ^Reference category for all comparisons - alcohol intake response "Never"

The proportions of preterm birth (<37 weeks), very preterm birth (<32 weeks), low birth weight (<2500 g) and birth weight below the 10^th ^centile were highest among women with a high consumption of alcohol. (Table 3) Of the very preterm births in the high alcohol intake group all (100%) were spontaneous preterm labours compared to the never drinkers where 49% were spontaneous preterm labours and 51% indicated preterm births due to obstetric complications. There were three cases of Fetal Alcohol Syndrome recorded in the entire cohort; one case in each of the groups of low, moderate and high intake. The proportion of infants born with congenital abnormalities and dysmorphic features was similar across all four groups of women. High alcohol consumption was associated with very preterm birth even after adjusting for socio-demographic confounding factors, adjusted OR 3.15 (95% CI 1.26 to 7.88) (Table 4). Adverse perinatal outcomes were not statistically increased in low and moderate drinkers compared to non-drinkers.

**Table 3:** Perinatal outcomes according to alcohol intake peri-conceptionally

Alcohol intake^i^	Never n = 11613 (%)	Low (0-5 units) n = 43455 (%)	Moderate (6 - 20 units) n = 6059 (%)	High (>20 units) n = 114 (%)
Gestational age (weeks), median (IQR)	39.9 (38.9 - 40.9)	40.1 (39.1 - 41.0)	40.1 (39.3 - 41.1)	40.1 (39.0 - 41.4)
Birth weight (grams), median (IQR)	3420 (3080 - 3765)	3520 (3175 - 3850)	3490 (3150 - 3810)	3345 (2865 - 3729)
Preterm birth <37 weeks	811 (7.0)	2215 (5.1)	304 (5.0)	13 (11.5)
Very preterm birth <32 weeks	161 (1.4)	438 (1.0)	63 (1.1)	6 (5.3)
Low birth weight <2500 g	619 (5.4)	1766 (4.1)	268 (4.5)	12 (10.7)
Very low birth weight <1000 g	51 (0.4)	162 (0.4%)	27 (0.5)	2 (0.8)
Birth weight <10^th ^centile	1622 (14.2)	5985 (14.0)	906 (15.2)	35 (31.0)
Male infant	5802 (50.8)	21813 (51.1)	3085 (51.7)	60 (53.1)
Perinatal death	97 (0.8)	313 (0.7)	47 (0.8)	2 (1.8)
Apgar score <3 at 1 minute	140 (1.2)	417 (1.0)	68 (1.1)	2 (1.8)
Apgar score <7 at 5 minutes	154 (1.3)	443 (1.0)	71 (1.2)	2 (1.8)
Admitted to neonatal unit	1799 (15.7)	6100 (14.3)	897 (15.0)	23 (20.4)
Congenital abnormality (any)	373 (3.3)	1192 (2.8)	197 (3.3)	3 (2.7)
Aneuploidy (Trisomy 21/13/18, XO)	13 (0.1)	57 (0.1)	6 (0.1)	1 (0.9)
Fetal Alcohol syndrome	0 (0.0)	1 (0.0)	1 (0.0)	1 (0.9)
Dysmorphic features (unclassified)	8 (0.1)	30 (0.1)	5 (0.1)	0 (0.0)

^i ^Reference category for all comparisons - alcohol intake response "Never"

**Table 4:** Risk of adverse perinatal outcomes in relation to alcohol intake peri-conceptionally

Alcohol intake^i^	Low (0 - 5 units) Unadjusted OR (95% CI)	Low (0-5 units) Adjusted OR^ii^ (95% CI)	Moderate (6 - 20 units) Unadjusted OR (95% CI)	Moderate (6-20 units) Adjusted OR^ii^ (95% CI)	High (>20 units) Unadjusted OR (95% CI)	High (>20 units) Adjusted OR^ii^ (95% CI)
Preterm birth <37 weeks	0.72 (0.66-0.78)	0.77 (0.69-0.85)	0.70 (0.61-0.81)	0.71 (0.60-0.83)	1.70 (0.55-3.05)	1.00 (0.53-1.87)
Very preterm birth <32 wks	0.76 (0.63-0.92)	0.88 (0.70-1.11)	0.82 (0.61-1.10)	0.95 (0.66-1.37)	3.93 (1.70-9.06)*	3.15 (1.26-7.88)*
Low birth weight <2500 g	0.75 (0.69-0.83)	0.69 (0.61-0.78)	0.82 (0.71-0.95)	0.71 (0.58-0.87)	2.09 (1.14-3.83)*	1.15 (0.60-2.22)
Birth weight <10^th ^centile	0.97 (0.92-1.03)	1.01 (0.96-1.06)	1.06 (0.98-1.17)	1.00 (0.92-1.09)	2.82 (1.88-4.23)*	1.44 (0.91-2.25)
Perinatal death	0.86 (0.69-1.08)	0.65 (0.47-0.90)	0.93 (0.65-1.32)	0.72 (0.44-1.18)	2.11 (0.51-8.65)	1.56 (0.33-7.44)
Apgar score <7 at 5 minutes	0.77 (0.64-0.92)	0.67 (0.53-0.85)	0.88 (0.66-1.17)	0.62 (0.42-0.92)	1.32 (0.32-5.41)	1.09 (0.25-4.73)
Admission to neonatal unit	0.89 (0.84-0.95)	0.84 (0.78-0.90)	0.95 (0.87-1.03)	0.83 (0.73-0.93)	1.37 (0.86-2.17)	0.91 (0.56-1.49)
Congenital abnormality (any)	0.85 (0.76-1.24)	1.01 (0.71-1.44)	1.01 (0.85-1.21)	1.01 (0.82-1.27)	0.81 (0.26-2.56)	0.56 (0.17-1.88)

^i ^Reference category for all comparisons - alcohol intake response "Never"

^ii ^adjusted for maternal age, single marital status, socio-economic status, nationality, private health insurance, nulliparity, unplanned pregnancy, late booking, smoking and history of illicit drug use

* p < 0.05

## Discussion

### Summary of main findings

This study found that a large number of women booking for antenatal care are consuming alcohol in the peri-conceptional period. Moderate alcohol intake was associated with Irish nationality, being in employment, smoking and having private health care. Heavy alcohol intake was associated with younger maternal age (less than 25 years) and illicit drug use. High alcohol intake was associated with preterm birth, very preterm birth and low birth weight on univariate analysis. The association with very preterm birth remained after controlling for socio-demographic confounding factors. The reported incidence of Fetal Alcohol Syndrome was lower than expected.

### Strengths and limitations of the study

The population consisted of a complete geographical cohort of women attending a large urban maternity hospital over an eight year period. The data were collected prospectively and ascertained routinely by a qualified midwife using a structured electronic database. The data on alcohol intake relied on self-reporting by the pregnant woman and it is likely that the amount of alcohol consumed is under-reported. It is unlikely that alcohol intake is over-estimated. Alcohol intake is recorded in bands rather than in actual grams or units of alcohol which limits the ability to assess a dose-response effect. There may also be a lack of understanding among women as to what constitutes a unit of alcohol (a small glass of wine, half pint of beer or small measure of spirits). As the data were collected at the booking visit (typically between 12 and 20 weeks gestation), the potential for recall bias was limited. The confounding factors were noted to have possible effects on perinatal outcomes in both directions (positive and negative) i.e. women with low and moderate intake were more likely to be in employment and have private health insurance whereas non-drinkers were more likely to be from ethnic minority groups and unemployed. We considered defining the low alcohol intake group as the reference category, however as there was one case of FAS in this group we thought it more appropriate to use the "never" group as the comparator particularly as "never" use is likely to be accurately reported. We did not have data on alcohol intake later in pregnancy and it is possible that maternal alcohol intake alters as pregnancy progresses. Neonates were examined prior to first hospital discharge and longer term follow-up would be required to detect all cases of fetal alcohol syndrome, fetal alcohol spectrum disorder and neuro-developmental deficit.

### Comparison with existing literature

The reported incidence of FAS was lower than expected given that the estimated incidence is 0.4 per 1000 live births for European countries [8]. There was one case of FAS in each of the groups of low and moderate intake as well as one case in the high intake group. This suggests that the mothers of the first two infants may have under-reported their alcohol intake at the time of booking as FAS is unlikely to occur at lower levels of alcohol intake. The low incidence of FAS in the cohort suggests that this syndrome is under-recognised prior to first hospital discharge and that increased awareness is needed to improve diagnosis, both in the neonatal period and in the older child.

It is also difficult to identify binge-drinkers from the data collected as women were not specifically questioned about their drinking patterns and many women who binge-drink may have a low to moderate intake overall. In one Danish study 50% of pregnant women reported at least one episode of binge-drinking during the time from their last menstrual period until pregnancy recognition [14,15]. Our study showed that heavy drinking in the peri-conceptional period was associated with maternal age less than 25 years. Binge drinking is more common in this age-group. It has been suggested that binge-drinking may do greater harm to the developing fetus than drinking a comparable amount over a longer period of time as peak blood alcohol concentration is the critical factor [12,16]. Public health campaigns will need to address the high rate of binge drinking among young women and the association with unplanned pregnancy and late booking for antenatal care.

Henderson et al performed a systematic review of the effects of low to moderate prenatal alcohol exposure on pregnancy outcome [2]. In keeping with our study they found no consistent evidence of adverse perinatal effects at this level of consumption. Since then O' Leary et al reported a cohort study investigating the effect of maternal alcohol intake on preterm birth [1]. The investigators collected data for the three-month period pre-pregnancy and for each trimester separately, however the data were collected postpartum and relied on recall. They also included a category for binge-drinkers. In keeping with our study, they found that the rate of low birth weight infants was highest among those exposed to either binge or heavy drinking in pregnancy. The rate of preterm birth was also highest among those infants exposed to binge-drinking and for infants of mothers who drank heavily but stopped drinking before the second trimester. A cohort study carried out by Albertsen et al [17] showed that an alcohol intake of seven or more drinks (84 g of alcohol) per week was associated with an increased risk of preterm birth and a prospective cohort study by Sokol et al [18] demonstrated a dose-response association between alcohol intake and extreme preterm delivery. As with our study, the risk of very preterm birth appeared to be even higher than the risk of moderate preterm birth.

### Implications for practice

Current government policies in Ireland, the United Kingdom and the United States of America recommend that it is in a child's best interests for women not to consume any alcohol during pregnancy [13,18]. It is clear from this study that large numbers of women are drinking in the peri-conceptional period, some at high levels, despite these warnings. There is a need for more accurate documentation of alcohol consumption so that at-risk drinkers can be identified early in pregnancy (ideally pre-conceptionally) and offered appropriate interventions to reduce the risk to the developing fetus. Very preterm birth is one of the main causes of neonatal mortality, morbidity and functional impairment. The prevention of high-risk drinking in pregnancy could reduce adverse perinatal outcomes with implications for the affected families and cost to the healthcare system. As it is likely that Fetal Alcohol Syndrome is under-recognised, babies born to women who drink alcohol during pregnancy should be followed in order to more accurately determine the incidence of Fetal Alcohol Syndrome and Fetal Alcohol Spectrum Disorder in this population. As there is continuing uncertainty regarding the effects of low levels of alcohol intake in pregnancy, more research related to this type of drinking is required.

## Conclusions

This study emphasises the need for improved detection and management of alcohol misuse in pregnancy and for early intervention in order to minimise the risks to the developing fetus. Further research is required specifically addressing the effects of low alcohol intake in pregnancy before it can be considered safe. Public health campaigns need to educate and change attitudes towards pregnancy and promote healthy lifestyle choices in women of child bearing age. An ideal approach would incorporate advice on diet, exercise, alcohol, smoking, drugs and folic acid supplementation as part of a pre-pregnancy planning strategy.

## Competing interests

The authors declare that they have no competing interests.

## Authors' contributions

DJM (guarantor) had the original idea for the study and, with all co-authors carried out the design. DJM and JB obtained funding. AM and BC were responsible for data cleaning. DJM, AM and BC carried out the analyses. AM and DJM drafted the manuscript which was revised by all authors. All authors read and approved the final manuscript.

## Pre-publication history

The pre-publication history for this paper can be accessed here:

http://www.biomedcentral.com/1471-2393/11/27/prepub
